# The association between residential eviction and syringe sharing among a prospective cohort of street-involved youth

**DOI:** 10.1186/s12954-017-0150-5

**Published:** 2017-05-12

**Authors:** Andreas Pilarinos, Mary Clare Kennedy, Ryan McNeil, Huiru Dong, Thomas Kerr, Kora DeBeck

**Affiliations:** 1 BC Centre on Substance Use, British Columbia Centre for Excellence in HIV/AIDS, 608-1081 Burrard Street, Vancouver, BC V6Z 1Y6 Canada; 2 Interdisciplinary Studies Graduate Program, University of British Columbia, 270-2357 Main Mall, Vancouver, BC V6T 1Z4 Canada; 3 School of Population and Public Health, University of British Columbia, 2206 East Mall, Vancouver, BC V6T 1Z3 Canada; 4 Division of AIDS, Department of Medicine, University of British Columbia, 667-1081 Burrard Street, Vancouver, BC V6Z 1Y6 Canada; 5 School of Public Policy, Simon Fraser University, 3277-515 Hastings W Street, Vancouver, BC V6B 5K3 Canada

**Keywords:** Eviction, Street-involved youth, Syringe sharing, Housing policy

## Abstract

**Background:**

Syringe sharing is a high-risk practice associated with the transmission of infectious diseases, such as HIV and HCV. While youth who contend with housing instability are known to be more likely to engage in high-risk substance use, the potential relationship between being evicted from housing and syringe sharing has not been examined. This study assessed whether residential eviction was associated with syringe sharing among street-involved youth in Vancouver, Canada.

**Methods:**

Data were derived from the At-Risk Youth Study (ARYS), a prospective cohort of street-involved youth who use drugs age 14–26 in Vancouver, Canada. The study period was June 2007 to May 2014, and the potential relationship between residential eviction and syringe sharing was analyzed using multivariable generalized estimating equations (GEE) logistic regression.

**Results:**

Among 405 street-involved youth who injected drugs, 149 (36.8%) reported syringe sharing, defined as borrowing or lending a syringe, at some point during the study period. In a multivariable GEE analysis, recent residential eviction remained independently associated with syringe sharing (adjusted odds ratio (AOR) = 1.72, 95% confidence interval (CI): 1.16–2.57), after adjusting for potential confounders.

**Conclusions:**

Syringe sharing was significantly elevated among youth who had recently been evicted from housing. These findings indicate that policy and programmatic interventions that increase housing stability may help mitigate high-risk substance use practices among vulnerable youth.

## Background

Injection drug use constitutes a significant public health concern due to its association with the transmission of blood-borne pathogens such as HIV and HCV as well as other severe health-related harms [1–4]. These negative health consequences are particularly pronounced among people who inject drugs (PWID) and share syringes [5]. Previous research has identified risk factors associated with syringe sharing that include difficulty accessing needles, binge drug use, injection cocaine use, and homelessness [6, 7]. While evidence suggests that there have been significant reductions in syringe sharing among adult populations in Vancouver, Canada, [8–10], research among street-involved youth indicates that rates of syringe sharing have persisted at concerning levels [11, 12].

Previous studies of street-involved populations have documented numerous health-related risks and harms associated with housing insecurity. To date, this work has predominantly focused on the adverse health impacts of homelessness, including increased likelihood of drug-related risk behaviors such as intensified substance use [13, 14], initiation of injection drug use [15, 16], public injecting [2], and syringe sharing [17]. More recently, there has been increased research interest in the health consequences of residential eviction (i.e., forced displacement of a tenant from a leased residence through legal or extra-legal mechanisms). For instance, eviction has been associated with an increased likelihood of experiencing violence [18] and exhibiting a detectable HIV-1 RNA viral load among adult PWID in Vancouver, Canada [19]. In addition, studies of other marginalized populations have demonstrated associations between housing displacement and other mental and physical health concerns including depression, anxiety, suicide, high blood pressure, and poorer self-rated health status [20]. At present, however, we know of no existing studies that have investigated the relationship between residential eviction and syringe sharing. Therefore, we sought to examine whether residential eviction was associated with syringe sharing among a prospective cohort of street-involved youth in Vancouver, Canada.

## Methods

This study is based on data from the At-Risk Youth Study (ARYS), an open prospective cohort of street-involved youth in Vancouver, Canada, that began in 2005. The study design of this prospective cohort has been previously described in detail [21]. In brief, recruitment consists of street-based outreach and snowball sampling. Eligibility is restricted to street-involved youth who are age 14–26 years at baseline, have used illicit drugs other than or in addition to marijuana in the past 30 days, and who provide written and informed consent. The term “street-involved” refers to youth who are currently or recently homeless or who recently accessed services for homeless youth. Upon enrolment, and bi-annually thereafter, an interview-administered questionnaire is conducted with participants and includes demographic information, drug use patterns, and practices, as well as engagement in health and social services. A stipend ($30 CAD) is provided to participants at each study visit. This study has been approved by the University of British Columbia and the Providence Health Care Research Ethics Board.

The study period for the present analysis was restricted to between June 2007 and May 2014, as the measures for residential eviction were only available during this period. To examine the potential relationship between residential eviction and syringe sharing, all analyses were restricted to study observations in which participants reported injection drug use in the last 6 months. The primary outcome of interest for this analysis was syringe sharing, defined as responding affirmatively to the question: “In the last 6 months, have you fixed with a rig that had already been used by someone else?” or “In the last 6 months have you lent your used rig to someone else?” (yes vs. no). The primary explanatory variable of interest was recent residential eviction, defined as responding affirmatively to the question: “Have you been evicted in the last 6 months” (yes vs. no).

To determine whether there was a significant relationship between our outcome of interest and our primary explanatory variable, we *a priori* selected other secondary explanatory factors we hypothesized might be associated with both residential eviction and syringe sharing. Secondary explanatory factors included: age (per year older), gender (female vs. male), ethnicity (Aboriginal Ancestry vs. other), binge drug use (yes vs. no), daily heroin use (yes vs. no), daily cocaine use (yes vs. no), daily crystal methamphetamine use (yes vs. no), difficulty accessing syringes (yes vs. no), public injecting (yes vs. no), incarceration (yes vs. no), and accessing drug treatment (e.g., pharmacotherapy, residential treatment, and counseling) (yes vs. no). All variables, excluding age, gender, and Aboriginal Ancestry, refer to circumstances and behaviors over the previous 6 months and were treated as time-updated covariates on the basis of semiannual follow-up data.

Initially, we examined the descriptive characteristics, stratified by reports of syringe sharing at the first study visit. Comparisons were made using the Pearson’s χ^2^ test for binary variables (Fisher’s exact test when cell counts were less than or equal to 5) and the Wilcoxon rank-sum test for continuous variables.

Next, we used generalized estimating equation (GEE) analyses with logit link function to assess the independent association between residential eviction and syringe sharing. These methods provide standard errors adjusted by multiple observations per person using an exchangeable working correlation structure [22, 23]. Therefore, data from every participant follow-up visit was considered.

To examine the associations between syringe sharing and each explanatory variable, we first conducted bivariate GEE analyses. To fit the multivariable model, we employed a conservative variable selection approach [24]. Specifically, we initially included the primary explanatory variable and all secondary variables where *p <* 0.10 in bivariate analyses in a multivariable model. We then used a stepwise approach to fit a series of reduced models. After comparing the value of the coefficient associated with the main independent variable of interest (residential eviction) in the full model to the value of the coefficient in each of the reduced models, we dropped the secondary variable associated with the smallest relative change. We continued this iterative process until the minimum change exceeded 5%. Remaining variables were considered confounders in the final multivariable model. *P* values are all two-sided. Statistical analyses were conducted using SAS software version 9.4 (SAS, Cary, NC).

## Results

Among 938 street-involved, 405 (43.2%) reported injection drug use over the study period. Of these, 142 (35.1%) were female and 91 (22.5%) reported being of Aboriginal Ancestry. The median age of the sample was 22.7 years (inter-quartile range (IQR) = 20.9–24.4]. This sample contributed 1131 observations, and the median number of study visits per participant during the study period that included a report of active injection drug use was 2 (IQR = 1–3). The median follow-up time per participant was 19.2 months (IQR = 6.6–48.3).

In total, 149 (36.8%) youth reported syringe sharing at some point during the study period and a total of 208 (18.4%) observations included a report of syringe sharing. Additionally, 114 (28.1%) unique participants in our sample reported being evicted at least once over the study period. Of these, 86 (75.4%) reported one eviction event, 19 (16.7%) reported two evictions, 7 (6.1%) reported three evictions, and 2 (1.8%) reported four evictions during follow-up. Among the 533 (56.8%) participants excluded from the study, the proportion who experienced residential eviction was not significantly different from those who were included in the study (12.2 vs. 13.5%, *P* = 0.304).

The baseline characteristics, stratified by syringe sharing in the last 6 months, are presented in Table 1. The results of the bivariate and multivariable GEE analyses are presented in Table 2. In bivariate analyses, recent residential eviction (odds ratio (OR) = 1.78; 95% confidence interval (CI): 1.20–2.65) was positively associated with syringe sharing. In the multivariable analyses, recent residential eviction (adjusted odds ratio (AOR) = 1.72, 95% confidence interval (CI): 1.16–2.57) remained significantly and positively associated with syringe sharing after adjusting for Aboriginal Ancestry, which was the only identified confounder.

**Table 1:** Baseline characteristics of street-involved youth in Vancouver, Canada, stratified by syringe sharing (*n* = 405)

Characteristic	Total (%) (*n* = 405)	Syringe sharing^a^		Odds Ratio *(95% CI)*	
		Yes (*%*) (*n* = 95)	No (%) (*n* = 310)		*p* value
Age (median, IQR)	22.7 (20.9–24.4)	22.2 (20.4–23.7)	22.9 (21.1–24.5)	–	0.055^a^
Female gender	142 (35.1)	37 (38.9)	105 (33. 9)	1.25 (0.77, 2.00)	0.364
Aboriginal Ancestry	91 (22.5)	12 (12.6)	79 (25.5)	0.43 (0.22, 0.83)	0.010
Binge drug use^b^	224 (55.3)	63 (66.3)	161 (51.9)	1.81 (1.12, 2.93)	0.015
Daily heroin use^b^	91 (22.5)	27 (28.4)	64 (20.6)	1.52 (0.90, 2.57)	0.116
Daily cocaine use^b^	9 (2.2)	2 (2.1)	7 (2.3)	0.93 (0.19, 4.54)	1.000^†^
Daily crytsal methamphetamine use^b^	79 (19.5)	19 (20.0)	60 (19.4)	1.04 (0.59, 1.85)	0.890
Residential eviction^b^	67 (16.5)	21 (22.1)	46 (14.8)	1.62 (0.91, 2.90)	0.099
Difficulty accessing syringes^b^	101 (24.9)	28 (29.5)	73 (23.5)	1.37 (0.82, 2.30)	0.225
Public injecting^b^	274 (67.7)	77 (81.1)	197 (63.6)	2.55 (1.44, 4.53)	0.001
Incarceration^b^	93 (23.0)	20 (21.1)	73 (23.6)	0.87 (0.50, 1.51)	0.613
Accessing drug treatment^b, c^	171 (42.2)	50 (52.6)	121 (39.0)	1.75 (1.10, 2.78)	0.018

†*p* value is generated from Fisher’s Exact Test because of small cell count

^a^Refers to continuous variable, *p* value is generated from Wilcoxon rank-sum test

^b^Refers to activities in the last 6 months

^c^Excludes detoxification services

**Table 2:** Bivariate and multivariable generalized estimating equation (GEE) analyses of factors associated with syringe sharing among street-involved youth in Vancouver, Canada, (*n* = 405)

	Unadjusted		Adjusted	
Characteristic	Odds ratio (95% CI)	*p* value	Odds ratio (95% CI)	*p* value
Residential eviction^a,b^	1.78 (1.20, 2.65)	0.004	1.72 (1.16, 2.57)	0.007
Age (per year older)	0.90 (0.84, 0.96)	0.001		
Female gender^b^	1.09 (0.75, 1.58)	0.656		
Aboriginal Ancestry^b^	0.51 (0.33, 0.80)	0.003	0.50 (0.32, 0.80)	0.003
Binge drug use^a,b^	1.58 (1.21, 2.07)	<0.001		
Daily heroin use^a,b^	0.93 (0.68, 1.25)	0.619		
Daily cocaine use^a,b^	0.89 (0.38, 2.10)	0.793		
Daily crystal methamphetamine use^a,b^	0.86 (0.57, 1.30)	0.471		
Difficulty accessing syringes^a,b^	2.11 (1.51, 2.94)	<0.001		
Public injecting^a,b^	2.88 (1.95, 4.25)	<0.001		
Incarceration^a,b^	1.26 (0.88, 1.80)	0.214		
Accessing drug treatment^a,b,c^	1.09 (0.80, 1.48)	0.589		

^a^Refers to activities in the last 6 months

^b^Comparison is yes vs. no

^c^Excludes detoxification services

## Discussion

Among this community-recruited cohort of 405 drug-using street-involved youth in Vancouver, Canada, syringe sharing was common with over one-third of study participants reporting borrowing or lending used syringes during the study period. In multivariable analyses, residential eviction was independently associated with syringe sharing.

Whereas existing literature has demonstrated that eviction may have harmful health-related consequences for drug-using populations [18–20] and that homeless and unstably housed street-involved youth experience higher rates of risky substance use [13, 25], the current study expands on this area of research by demonstrating that experiencing residential eviction is linked with an increased likelihood of syringe sharing. One potential explanation for this finding is that evicted street-involved youth may be displaced to other neighborhoods [26], reducing spatial access to services that provide sterile injecting equipment. Additionally, youth who are displaced due to residential eviction may subsequently experience homelessness, which has been positively associated with syringe sharing [7]. For example, homeless individuals may be more likely to come into contact with police or experience violence, which may result in the loss of sterile injecting equipment and an increased likelihood of sharing syringes [27–29].

Another possible explanation is that evicted street-involved youth may subsequently become homeless or unstably housed [30–32] and, as a result, may be more likely to use drugs in public settings, which has previously been associated with syringe sharing [33]. However, given that the analyses employed herein limit interpretation of temporal relationships, it could also be that street-involved youth who share syringes are more heavily involved in the street-based drug scene or are more likely to experience financial instability, which might increase their vulnerability to residential eviction [32]. Further research is needed to better understand the mechanisms underlying the observed association between residential eviction and syringe sharing.

While accessing housing remains imperative to reducing risky substance use practices among youth, the findings of the present study suggest that maintaining housing stability may also play an important role in reducing such practices. Previous research among street-involved youth in this setting indicate that financial instability is among the most commonly reported reasons for eviction [32]. A recent study also found that 52% of street-involved youth in Vancouver reported being unable to access housing and housing referral services [34]. The current study findings extend this work and underscore the importance of ensuring that youth receive necessary social and financial supports to access and maintain housing in order to prevent eviction and reduce risky substance use behaviors.

Given that over one-third of study participants reported syringe sharing at some point during the study period, it is evident that interventions that reduce syringe sharing among street-involved youth are needed, particularly among those experiencing residential eviction. Among adult populations with concurrent disorders, existing literature has pointed to the effectiveness of providing housing supports. Examples of housing supports include access to case management supports, home support services, crisis intervention services, and health care services [35, 36]. The provision of housing supports have been associated with significant reductions in substance use and risky practices [34, 37–39], as well as improved housing stability among street-involved populations [40–42].

While providing housing supports has realized significant successes among adult populations and has been integrated into youth homelessness strategies in the USA, Australia, and the UK, Canada has been slow to implement such a model for street-involved youth [43]. Therefore, expanding housing options and supports for youth may have the potential to reduce experiences of eviction and related risky substance use practices among this population. Particular emphasis should be placed on ensuring that housing options espouse a continuum of care that ranges from low-threshold to abstinence-based housing in order to meet diverse range of housing needs facing marginalized populations of youth, particularly those who inject drugs.

This study has a number of limitations. First, because self-reported responses were solicited for this survey, results are subject to response biases. Existing literature, however, suggests that self-reported responses among street-involved populations are generally reflective of actual behaviors [44]. Nonetheless, we suspect that socially desirable responses would likely lead to an underreporting of high-risk practices thereby potentially leading to conservative estimates of syringe sharing. Second, the ARYS cohort is a non-randomized, community-recruited sample of street-involved youth in Vancouver and therefore may not be generalizable to other populations. Third, given the observational nature of the study, there may be unmeasured confounding. In addition, the methods employed herein did not allow us to determine the temporality of the association between residential eviction and syringe sharing.

## Conclusions

In sum, this study found that youth who recently experienced residential eviction had a significantly higher likelihood of sharing syringes. These findings suggest that policy approaches aiming to provide youth with access to a continuum of housing supports may have significant potential to mitigate residential eviction and high-risk substance use among street-involved youth.

## Abbreviations

ARYS: At-Risk Youth Study
PWID: People who inject drugs
